# MicroRNA Profiling of the Pathophysiological Mechanisms Underlying Carotid Atherosclerosis Progression

**DOI:** 10.3390/biom16071026

**Published:** 2026-07-14

**Authors:** Katarzyna Prus, Marco Sanvitti, Federico Bilotta

**Affiliations:** 1 Department of Stroke and Early Post-stroke Rehabilitation, University Clinical Hospital No. 4, 20-090 Lublin, Poland; katarzyna.prus@usk4.lublin.pl; 2 Department of Pediatric Surgery, “Sapienza” University of Rome, 00185 Rome, Italy; sanvitti.1927256@studenti.uniroma1.it; 3 Department of Anesthesiology, Intensive Care and Pain Medicine, University of Tor Vergata, 00133 Rome, Italy

**Keywords:** biomarkers, carotid stenosis, endothelial dysfunction, microRNAs, plaque, atherosclerotic, vascular smooth muscle cells

## Abstract

Carotid atherosclerosis is a major contributor to ischemic stroke, driven by complex interactions between inflammation, lipid metabolism, and vascular dysfunction. MicroRNAs (miRNAs) have emerged as key regulators of the molecular mechanisms underlying plaque formation, progression, and instability. This review summarizes current evidence on the role of miRNAs in endothelial dysfunction, vascular smooth muscle cell proliferation, macrophage activation, and plaque vulnerability. Specific miRNAs, including miR-21, miR-145, and miR-200c, demonstrate context-dependent effects on atherogenic and protective pathways. Circulating miRNA profiles show strong potential as noninvasive biomarkers for assessing plaque instability and predicting cardiovascular ischemic events, and as promising therapeutic targets. Despite these advances, challenges remain in standardization and clinical implementation. Integration of multi-miRNA panels with imaging modalities, conventional biomarkers, and emerging artificial intelligence approaches may improve risk stratification and support personalized management of carotid artery disease.

## 1. Introduction

Carotid atherosclerotic disease (CAD) is a well-established risk factor for ischemic stroke [1]. Understanding the etiopathology of plaque formation and stenosis progression is essential for managing patients with carotid stenosis and anticipating complications [2]. Atherosclerotic plaque formation begins at the abluminal surface, where metabolic, mechanical, or physical injury disrupts endothelial integrity. This chronic process leads to pathological lipid deposition, platelet aggregation, and thrombus formation, which can result in stenosis or vessel occlusion. MicroRNAs are emerging as important bioregulators of atherosclerotic plaque development [3]. They affect key stages of carotid artery disease, including endothelial dysfunction, vascular smooth cell proliferation, monocyte recruitment and foam cell formation, and plaque instability. MiRNAs are functionally involved in the atherosclerotic process by modulating gene expression, affecting endothelial dysfunction, enabling VSMC phenotypic switching, macrophage polarization, and oxidized lipid (oxLDL) efflux, which are fundamental to plaque formation, progression, and destabilization [4].

Various complex mechanisms contribute to the pathogenesis of carotid atherosclerosis, with inflammation and the body’s immune response playing key roles [5]. Numerous inflammatory mediators, along with other indicators of pathological processes, have been proposed as potential biomarkers of atherosclerosis. Currently, only serum lipoprotein-associated phospholipase A2 (Lp-PLA2) and high-sensitivity C-reactive protein (hs-CRP) concentrations are FDA-recommended for cardiovascular risk assessment in the course of carotid disease [6]. However, to date, standardized laboratory tests have demonstrated high specificity for predicting progression of carotid atherosclerotic disease or cardiovascular ischemic events (CIEs). Recently, there has been increased focus on miRNA fragments as key players in the pathogenesis of atherosclerosis. Research confirms that miRNA expression correlates with cholesterol metabolism, lipid oxidation, endothelial dysfunction, and inflammation during plaque formation and evolution [7,8,9]. Additionally, it affects vascular smooth muscle cell (VSMC) proliferation and the stabilization of atherosclerotic plaque. MiRNAs are notable for their high tissue specificity and stability across various body fluids and conditions, making them promising as both biomarkers of plaque vulnerability and potential therapeutic targets [10]. All of these features prompt a focused analysis on the involvement of miRNAs in the etiopathology and progression of carotid atherosclerotic disease.

The aim of this review is to present the available scientific data on the role of miRNAs in the pathogenesis of carotid atherosclerosis and carotid stenosis progression.

## 2. Role of Specific miRNAs in Etiopathology of Atherosclerosis

The miRNAs have been shown to regulate diverse cellular processes and are currently proposed as markers of pathological states [11,12]. The binding of miRNAs to different mRNA regions may result in both silencing and enhancing effects on gene expression. Fragments of miRNA can be secreted into extracellular fluids and transported to target cells via exosomes or bound to proteins. Specific miRNA expression patterns have been detected in body fluids (serum, plasma, CSF) of patients with atherosclerosis and in unstable atherosclerotic plaques [13]. Research data indicate that miRNA expression shifts occur across all cell types involved in the etiopathology of atherosclerosis, including endothelial cells (ECs), VSMCs, and macrophages [14]. The miRNAs reportedly coregulate different stages of pathologies underlying plaque formation and progression—endothelial dysfunction, smooth muscle cell differentiation, endothelial activation, monocyte recruitment, foam cell formation, and finally plaque evolution [15]. Researchers report that specific miRNA fragments may play both protective and destructive roles in the atherogenic process.

### 2.1. Endothelial Dysfunction

The endothelium is central to vascular homeostasis, and its pathologies play a major role in plaque formation [15]. Recent studies have demonstrated the critical role of ECs in the pathogenesis of atherosclerosis, demonstrating their involvement in immune cell signaling, oxidative stress, and other complex biological processes. Endothelial dysfunction is the initiating step in the development of atherosclerotic lesions. This early, reversible stage is characterized by reduced nitric oxide (NO) bioavailability, increased endothelial permeability, and chronic inflammation [16]. Further activation of ECs and platelets plays a crucial role in the development of atherosclerotic plaques and subsequent thrombotic disorders. This process is strongly associated with the formation of unstable, echolucent plaques and may therefore result in progression of carotid stenosis and occurrence of CIEs [17]. Research indicates that miRNA expression may be significantly correlated with both protective and pathological processes affecting the endothelium through various mechanisms. MiR-21 is involved in the endothelial responses to different shear stress patterns, acting as a mediator of both protective and pathological processes depending on the hemodynamic context [18]. Under pro-atherogenic, oscillatory, or low-shear stress conditions, miR-21 is upregulated and contributes to a maladaptive inflammatory endothelial transformation. Elevated miR-21 expression triggers Activator Protein-1, a critical regulator of EC function, leading to upregulation of endothelial adhesion molecules and chemokines. Conversely, under normal hemodynamic conditions, miR-21 appears to play a protective role in maintaining endothelial homeostasis by reducing endothelial apoptosis and promoting NO-mediated vasoprotection. Overexpression of some miRNA fragments affecting the function of ECs may be triggered by oxidized low-density lipoproteins. Available research data state that miR-92a plays a significant role in endothelial pathology by enhancing endothelial activation and atherosclerosis progression [19]. Upregulation of miR-92a by lipoproteins has been proven to induce endothelial dysfunction. Another study by Bonaire et al. confirmed that miR-92a overexpression leads to endothelial damage in cardiovascular ischemic diseases, whereas treatment with a miR-92a antagomir may improve vascular repair [20]. Furthermore, miR-92a expression was reported to be higher in patients with acute myocardial infarction than in those with stable coronary artery disease [21] and in patients with acute carotid stenosis than in healthy controls [22], thereby demonstrating its correlation with the risk of CIE.

One of the most detrimental factors affecting endothelial function is chronic inflammation. Available research data confirmed a correlation between the expression of specific miRNAs and inflammation-triggered endothelial dysfunction. A comparative study of atherosclerotic plaque stability demonstrated that miR-200c activity is positively correlated with the level of inflammatory markers, including monocyte chemoattractant protein-1, cyclooxygenase-2 (COX-2), interleukin 6 (IL-6), matrix metalloproteinase 1 (MMP-1), and matrix metalloproteinase 9 (MMP-9) [23]. At the same time, it was negatively correlated with protective agents indicated by zinc finger E-box binding homoeobox 1 (ZEB1), endothelial nitric oxide synthase (eNOS), forkhead boxO1 (FOXO1), and Sirtuin1 (SIRT1). Circulating miR-200c expression was upregulated before carotid endarterectomy (CEA) and decreased 1 day after CEA. Furthermore, research data confirm that miRNAs may affect ECs by regulating their barrier function in inflammation. A study by Reijerkerk et al. confirmed that miR-125a-5p plays a critical role in the formation of a tight brain endothelial barrier, reducing vascular permeability, lipid accumulation, and uncontrolled leukocyte transmigration [24]. Results demonstrated that cells with increased miR-125a-5p expression tend to form thicker, more continuous junctional complexes of VE-cadherin and zona occludens-1, whereas the opposite characteristics were observed in cells with reduced miR-125a-5p expression.

Endothelial dysfunction is also strongly associated with oxidative stress and the production of reactive oxygen species (ROS) [25]. Numerous miRNAs have been reported to regulate EC function by modulating cellular responses to redox imbalance. Research data confirm that oxidative stress-induced miR-200c expression directly targets SIRT1. SIRT1 upregulation is associated with beneficial effects on ECs, while excessive ROS production decreases SIRT1 expression, leading to endothelial dysfunction [26]. Other studies suggest that miR-210 regulates oxidative stress response by playing a key role in mitochondrial metabolism. The transcriptional activation of miR-210 can block both mitochondrial respiration and mitochondrial electron transport chain (ETC) activity by downregulating several steps of the mitochondrial metabolism, including repression of Iron–Sulfur Cluster Assembly Enzyme (ISCU), targeting essential components of the mitochondrial complex I, II, and IV, and NADH dehydrogenase [25]. MiRNAs can affect mitochondrial oxidative defense by decreasing the function of superoxide dismutase-2 (SOD-2). It was confirmed that miR-21 upregulation in atherosclerotic plaques activates the ERK/MAPK pathway, resulting in increased ROS formation and impaired migration of angiogenic progenitor cells [27].

EC dysfunction is strongly correlated with VSCM invasion into the intima. The miR-143/miR-145 cluster is reported to play an important role in the interaction between smooth muscle cells and endothelial cells [28]. Thus, it is considered a potential biomarker in atherosclerotic disease [29]. Accumulating evidence has proved a significant association between miRNA profile and endothelial pathologies in the course of CAD. Altered expression of miRNAs was also reported for the next important stage of atherosclerotic plaque formation, strongly related to endothelial dysfunction—the proliferation and differentiation of VSMCs.

### 2.2. Vascular Smooth Muscle Cells’ Proliferation and Differentiation

The smooth muscle cells of the tunica media are responsible for vessel wall integrity and blood flow control, yet they also play a central role in both the development of atherosclerosis and the rupture of plaques. During the early stage of atherosclerosis, medial VSMCs invade the intima, promoting plaque formation. In the later stages of the disease, VSMCs’ proliferation, migration, and apoptosis affect the stability of atherosclerotic plaques by strengthening the fibrous cap. Although plaque proliferation and migration can increase fibrous cap thickness, apoptosis accelerates plaque rupture and triggers cardiovascular complications. Autophagy in VSMCs during late-stage atherosclerosis can lead to cell death, thereby destabilizing late-stage plaques.

Numerous miRNA fragments have been shown to regulate VSMC proliferation, migration, and apoptosis during CAD [9,30,31]. Each miRNA can regulate the expression of multiple target genes, which are often involved in the same cellular pathway and may promote or inhibit VSMC proliferation. A study by Gao et al. demonstrated that upregulation of miR-195-5p promoted VSMC proliferation and migration, while downregulation had the opposite effect [30]. MiR-195-5p, as a molecular driver of phenotypic switching acting through p53 and MAPK signaling pathways, may contribute to the progression of carotid stenosis. Another miR directly associated with VSMC function is miR-532-3p. It exerts this function by targeting programmed cell death protein 4 (PDCD4), which regulates VSMC proliferation, migration, and apoptosis [32].

VSMCs participate in plaque stabilization and vascular remodeling, thereby playing a crucial role in the formation of atherosclerotic plaques [33]. During atherosclerosis progression, VSMCs undergo a phenotypic switch in which they lose expression of contractile markers and acquire synthetic, proliferative, or macrophage-like characteristics. Expression of VSMC contractile genes is regulated by a number of transcription factors, including noncoding RNAs. MiRNAs may, however, affect both lesion growth through pathological proliferation in the early stages of the disease and support the structural integrity of advanced plaques. miR-21, described as one of the best-characterized microRNAs, is involved in the etiology of atherosclerosis in a context-dependent manner [18]. In response to vascular injury or mechanical stress, miR-21 expression is upregulated in VSMCs, promoting their differentiation and abnormal proliferation. At the same time, in later stages of plaque formation, miR-21 promotes the synthesis of extracellular matrix proteins that reinforce the fibrous cap, and therefore contribute to plaque stability. MiRNAs exert their biological function by regulating the translation of numerous genes. A study by Wang et al. confirmed that miR-361-5p promotes VSMC proliferation and migration by targeting the tissue inhibitor of matrix metalloproteinase 4 (TIMP4) gene. TIMP4 is negatively associated with intima-media (IM) thickness, and its absence results in atherosclerotic plaque deposition [34].

MiRNAs involved in pathological processes affecting VSMCs have shown potential as predictors of CIEs. Research on the roles of miR-145 and miR-210 in CAD has confirmed that patients with low expression levels of these miRNAs have a higher risk of cerebral ischemia [8]. Both miRNAs play a significant role in maintaining plaque stability. While miR-145 plays a critical role in the early stages of CAD pathogenesis by regulating the phenotype of highly expressed VSMCs and inhibiting their transition to synthetic, proliferative, or migratory phenotypes, miR-210 directly targets the tumor suppressor adenomatous polyposis coli (APC) gene, promoting VSMC survival and reducing apoptosis. MiR-143, like miR-145, maintains the contractile-stable phenotype of VSMCs. Downregulation of miR-143 is associated with increased proliferation and migration of VSMCs in atherosclerotic plaques [31].

Researchers suggest that the expression of selected miRNAs involved in VSMC function may be strongly affected by metabolic disorders. Xing et al. demonstrated that not only was miR-206 expression lower in atherosclerotic tissue samples, but high blood glucose and lipid levels additionally decreased miR-206 expression by inducing metabolic inflammation [35]. These metabolically induced functional changes in VSMCs have also been reported to correlate with reduced relative survival of VSMCs and, subsequently, an increased risk of CIE [36].

MiRNA expression was also proven to be correlated with the next crucial element of the atherosclerotic process—macrophage infiltration and foam cell formation.

### 2.3. Monocyte Recruitment and Foam Cell Formation

Macrophages, together with ECs and VSMCs, are the main cell types involved in the pathophysiology of CAD, and evidence indicates that macrophage infiltration and VSMC proliferation are crucial in accelerating disease progression [37]. In the complex microenvironment of atherosclerotic plaques, macrophages are stimulated by diverse signals and exhibit a broad spectrum of activation states. Different monocyte subsets have been shown to contribute to this process in polarized ways. Resting macrophages exhibit multidirectional activity and can dynamically transition between pro- and anti-inflammatory states as well as form other subtypes engaged in hemoglobin clearance or antioxidant defense [38]. Depending on environmental signals, plaque-associated macrophages can exhibit both pro- and anti-atherogenic properties by modulating lipid metabolism, inflammation, and plaque stability [39]. Macrophages can exacerbate plaque instability by excessive secretion of pro-inflammatory cytokines, whereas plaque stability can be supported by increased efferocytosis, collagen deposition, and vascular repair.

There is substantial evidence of the significant impact of monocytic miRNAs on inflammatory responses and the etiology of atherosclerosis [38]. Canfran-Duque et al. reported that miR-21, the most abundant miRNA found in macrophages, limits the progression and promotes the regression of atherosclerosis [40]. Macrophage miR-21 expression strongly affects foam cell formation, sensitivity to ER-stress-induced apoptosis, and phagocytic clearance. Researchers state that miR-21 downregulation in macrophages promotes the induction of p38-CHOP and JNK signaling by targeting MKK3. This enhances macrophage apoptosis and promotes the post-translational degradation of ABCG1, a transporter that regulates macrophage cholesterol efflux. Intense proinflammatory cytokine secretion and severe hypoxia can also robustly increase miR-155 expression. Upregulated miR-155 amplifies inflammation by inhibiting STAT signaling. MiR-155 can also directly repress Bcl6, thereby promoting aggressive vascular inflammation, monocyte recruitment, foam cell formation, and inducing atherosclerotic plaque development [41]. Additionally, the miR-342-5p/Akt1 axis cooperates to enhance macrophage proinflammatory features, establishing a positive feedforward loop strictly dependent on miR-155 [42]. These interactions demonstrate how multiple miRNAs can synergize in enhancing the inflammatory processes underlying plaque formation. Conversely, miR-22-3p appears to reduce the inflammatory response in CAD by inducing the anti-inflammatory macrophage phenotype and thereby suppressing NLRP3 inflammasome activation via the Janus Kinase 1 (JAK1) gene [43]. It was reported that lower miR-22 levels may predict a higher incidence of recurrent ischemic stroke in patients with moderate carotid stenosis [44].

The polarization pathways are regulated by many other factors. Data indicate that miR-126 may improve efferocytosis and plaque stability, facilitating anti-inflammatory phenotype switching by targeting the vascular endothelial growth factor A (VEGFA)/KLF4 axis. miR-92a targets Krüppel-like factor 4 (KLF4) responsible for anti-inflammatory reprogramming. Its inhibition alleviates atherogenesis through targeting KLF4. Researchers suggest that neutralizing miR-92 may be a novel therapeutic strategy by reducing pro-inflammatory macrophage gene expression and increasing their anti-inflammatory activity [45]. Similarly, their pro-inflammatory counterparts frequently exert synergistic suppression, converging on shared pathogenic hubs and imposing negative feedback control to resolve inflammation. A cluster of miRNAs (miR-223, miR-146a, and miR-218-5p) reduces the signaling of the main initiator of pro-inflammatory cytokine release—Toll-like receptor 4 (TLR4), therefore inhibiting the secretion of TNF-α, IL-6, and IL-12 [46]. MiRNAs may affect the cytokine profile through MAPK signaling (e.g., miR-124) or by suppressing LPL (e.g., miR-152) [47,48].

MiRNAs may also affect macrophage lipid metabolism and accumulation at different stages. In recent studies, miR-27 and miR-329 have been shown to downregulate the expression of monocytic ATP-binding cassette (ABC) proteins [13]. ABCA1 and ABCG1 are essential in eliminating excess cholesterol and forming high-density lipoprotein, which contributes to the prevention and regression of atherosclerosis. Research data confirmed that the expression levels of miR-27 and miR-329 in mononuclear cells are inversely correlated to ABCA1 and ABCG1 gene expression in patients with confirmed CAD. Research data indicate that miR-26, miR-27a/b, miR-17-5p, miR-20a/b, miR-325, miR-19b, miR-33, miR-613, and miR-758 directly target ABCA1, and that miR-320b can modulate ABCG1 expression [38]. Lipid homeostasis in CAS is additionally affected by miR-144-3p and miR-134. These miRNAs suppress ABCA1 and ANGPTL4 (an LPL inhibitor), respectively, causing lipid accumulation and impaired cholesterol efflux. This directly triggers a feedforward loop that further amplifies the secretion of proinflammatory cytokines, TNF-α, and IL-6. MiRNA may also affect Stearoyl-CoA desaturase-1 expression, which regulates unsaturated fatty acid (UFA) levels in macrophages [49]. Liu et al. demonstrated that miR-127-3p overexpression could lead to decreased UFA content, increased acetyl-CoA content, and increased oxidative phosphorylation. Data indicate that miR-127-3p-overexpressing macrophages exhibit increased proliferation capacity and cholesterol accumulation, thereby promoting atherosclerosis formation and decreasing plaque stability [50]. Studies have confirmed that elevated miR-127-3p expression in human advanced atheroma was positively associated with macrophage accumulation in carotid plaques.

Macrophage apoptosis is a crucial process in CAD that prevents atherosclerotic plaque development in the early stages but, at the same time, reduces the stability of advanced atherosclerotic lesions. It can be triggered by various factors, including oxidative stress, hypoxia, pro-inflammatory cytokine secretion, and cholesterol overload. In the course of CAD, miRNAs are involved in both pro- and anti-apoptotic cascades and interact with diverse molecular targets [40,51]. Autophagy is another critical phase of macrophage life that implicates carotid plaque stability. MiRNAs have been shown to modulate key steps in autophagic flux, influence foam cell formation, and regulate inflammatory responses. MiRNAs regulating all the pathways mentioned above significantly contribute to the control of atherosclerotic plaque stability [38].

### 2.4. Plaque Instability

Plaque stability strongly depends on the balance between the fibrous cap and lipid core. Inflammatory cytokines may affect interstitial collagen synthesis, increase matrix degradation, and promote apoptosis, enhancing plaque vulnerability and risk of rupture [49]. Changes in the expression of multiple miRNAs play an active role in atherosclerotic plaque vulnerability. Numerous studies have confirmed that carotid plaque rupture, which can lead to an acute ischemic event, is associated with alterations in circulating miRNA profiles.

Identifying vulnerable plaques before they become unstable is crucial for preventing ischemic complications [4]. MiRNAs linked to plaque instability play a major role in vascular inflammation, oxidative stress, modulation of endothelial dysfunction, macrophage differentiation, and lipid uptake in oxLDL [52]. Huang et al. confirmed that miR-146a expression in peripheral blood monocytes from patients with vulnerable plaque correlates with IL-6 and TNF-*α* levels; however, the direct molecular target of this miRNA was not elucidated [53]. Results of this research demonstrated that miR-146a had strong predictive value for plaque vulnerability in patients with CAS. Its expression was higher in CAS patients than in healthy controls and positively correlated with the degree of stenosis. Another study confirmed that miR155 was elevated in pro-inflammatory macrophages within atherosclerotic lesions, suggesting its potential role in increasing plaque vulnerability by modulating the expression of inflammation-associated genes [54].

A significant factor defining plaque stability is its composition. The fibrous cap thickness is the primary determinant of plaque stability and rupture risk [55]. As mentioned before regarding miR-21, altered miRNA expression may enhance the structural stability of advanced plaques [18]. Research has demonstrated that both miR-206 and miR-638 act as plaque-stability protectors by preventing VSMC phenotype switching [36,56]. Their downregulation was significantly correlated with the elevated risk of CIE. Patients with symptomatic CAS demonstrate a higher prevalence of echolucent plaques compared to asymptomatic patients [2]. Unstable plaques are associated with intra-plaque hemorrhage, large necrotic cores, rupture of fibrous caps, and luminal thrombosis. A study by Badacz analyzed the miRNA profiles of CAS patients based on atherosclerotic plaque composition [57]. Results demonstrated that patients with soft and echolucent plaques had significantly altered serum levels of miR-124-3p, miR-134-5p, miR-34a-5p, miR-375, and miR-133b. Overexpression of miR-16-5p and miR-1-3p was characteristic of ulcerated plaques. High levels of miR-1-3p were an independent risk factor for cardiovascular diseases and myocardial infarction, and miR-133b levels were positively associated with the incidence of CIE.

Vulnerable plaques tend to exhibit miRNA/gene dysregulation at multiple levels. Macrophage-rich, vulnerable plaques are characterized by overexpression of granulocyte-macrophage colony-stimulating factor 2 (GM-CSF) receptor alpha subunit (CSF2RA), which is downregulated by miR-532-3p via binding to CSF2RA’s 3′UTR [58]. Unstable plaques demonstrate downregulation of Talin-1, an essential mediator of integrin activation that promotes cell proliferation by recruiting and activating focal adhesion proteins. These regulatory functions may play an important role in plaque formation and stability. miRNAs that directly regulate the expression of Talin-1 (miR-330-5p, miR-432-5p, miR-7150, and miR-4260) are reported to be overexpressed in unstable carotid plaques, with miR-330-5p showing the most significant difference compared with stable plaques [59].

The overall miRNA profile can provide additional information on the potential risk of CIE in patients with vulnerable carotid plaques. Clinical data show that the serum ratio of overexpressed to underexpressed miRNAs can differentiate between CAD patients with urgent symptoms and those with asymptomatic CAD [60]. Numerous miRNA fragments were found to be upregulated, including miR-23a-5p, miR-320a, miR-2110, and miR-134-5p, in vulnerable versus stable plaque patients, while lower expression of miR-4439 was confirmed in the vulnerable group [60]. Preliminary research confirmed that an assessment score based on a panel of circulating miRNAs may enable prognostication of elevated risk of plaque rupture and acute ischemic stroke [61]. These results strongly suggest the potential of miRNAs as biomarkers of CAD and stenosis progression.

## 3. Potential Role of miRNA as Biomarkers of CAD, Stenosis Progression and CIE Risk Assessment

Atherosclerosis is an important risk factor for CIE, being the cause of an estimated 10% of ischemic strokes [62]. Progression of carotid artery stenosis (CAS) often occurs without clinical symptoms. Identifying patients at risk of developing CIE is critical for implementing effective prevention strategies to potentially avoid the grave consequences of stroke or myocardial infarction [63]. Recently, it is believed that the degree of luminal stenosis is not the best predictive indicator of stroke risk, as the morphology of the plaque plays a significant role in the prediction of cardiovascular complications [64]. Up to 30% of ischemic strokes occur in patients with high-risk non-stenotic plaques characterized by thin soft caps, ruptured or ulcerated inflamed fibrous caps, intraplaque hemorrhage, active inflammation, and large lipid-rich necrotic cores [65]. Neuroimaging techniques such as transcranial Doppler, CT perfusion, computational fluid dynamics, and quantitative MRA are advancing, but the complexity of assessment and limited availability of the latest technologies limit their use in everyday practice [66]. There is a growing need for biomarkers of pathologies involved in stenosis progression and plaque instability.

Up-to-date AHA/ASA recommendations include monitoring only hs-CRP and Lp-PLA2 levels to evaluate increased risk of CIE in the course of carotid sclerosis [6]. These markers, although easily accessible, are not specific enough to provide a clear picture of the underlying pathologies. Fragments of miRNA, as sensitive and highly specific markers of particular mechanisms underlying CAD etiology, provide greater insight into plaque vulnerability and subsequent stroke risk. The intricate regulatory networks controlled by miRNAs have led to the identification of specific miRNA signatures—patterns of over- or underexpressed miRNAs that are uniquely associated with particular conditions [67]. In atherosclerosis, these signatures can differentiate between stable and unstable plaques, and therefore enable the prediction of rupture before the onset of stroke or myocardial infarction. Research data demonstrate that miRNAs regulate not only the well-known elements of the atherosclerotic process, such as endothelial activation, VSMC proliferation, and macrophage recruitment, but are also involved in angiogenesis and may promote vascular remodeling, thereby assisting in the development of cerebral collateral circulation [68]. That paves the way for even more promising future perspectives on the use of miRNAs in therapeutic decisions regarding the need for revascularization.

The progression of CAS is defined as the increase in arterial narrowing due to plaque growth. It is associated with an elevated risk of CIE [69]. Researchers confirmed that patients with stenosis progression demonstrated elevated levels of circulating miR-199b-3p, miR-130a-3p, and miR-24-3p [63]. MiRNA profiling of carotid plaques could serve as a prognostic tool for predicting stenosis progression, enabling identification of patients at high risk of CIE who could benefit from early revascularization. Available data confirm that selected miRNA levels are correlated with plaque stability and, therefore, with a higher risk of ischemic complications. Research results indicate that patients with unstable plaques exhibit higher levels of miR-200c, miR-127-3p, and miR-330-5p [25,50,59]. Numerous studies have confirmed the potential of miRNAs to predict the risk of CIE in CAS patients. Liu et al. confirmed the predictive value of miR-9-5p in a follow-up assessment of patients with CAD. Those with low miR-9-5p expression were more likely to undergo cardiovascular events [70]. In another study, miR-125a was an independent predictor of CIEs, along with severe stenosis and low HDL [7]. Analysis of miR-532-5p expression in CAS patients revealed a significant association with the degree of carotid stenosis in both symptomatic and asymptomatic patients [71]. The study results indicated that more CIEs were observed in asymptomatic patients with low serum levels of miR-532-5p. Down-regulation of serum miR-532-5p was also confirmed as an independent factor for the occurrence of CIEs in asymptomatic CAS. Another miRNA confirmed to be correlated with the incidence of cardiovascular complications in CAS patients is miR-92q. Research data showed that the patients with high serum miR-92a levels experienced more TIE and stroke episodes. Also, in this study, miR-92a appeared to be an independent risk factor for CIE in asymptomatic CAS [22]. In a latest study by Wen et al., researchers proposed miR-22 as a possible predictor of recurrent ischemic stroke (RIS) in CAS patients [44]. Results from this multicenter, prospective study revealed that patients with lower miR-22 levels had a higher risk of RIS. Although many miRNAs have been reported as independent markers of potential CIE and CAS progression, research indicates that panels of multiple miRNAs may outperform single-miRNA markers in assessing cerebrovascular risk [61,72]. It is also suggested that diagnostic and prognostic sensitivity and specificity of individual biomarkers of vulnerable carotid plaque may be improved by combining tissue and circulating miRNA signatures, lipid profiles, and protein and inflammatory biomarkers [73,74,75]. The latest advances in artificial intelligence (AI) give a new perspective on tailored protocols for biomarker discovery, including machine learning algorithms [76]. The technique can incorporate sociodemographic, clinical, and pharmacological information to discriminate between cases and controls or to predict disease risk or outcome. Thanks to AI, it is possible to weight and combine the values of several genes to produce a miRNA signature with predictive potential. In CAD, another promising aspect of incorporating AI into the diagnostic process may be the integration of laboratory biomarkers, clinical data, and carotid and atherosclerotic plaque imaging [77]. Precise identification of unstable carotid plaques through non-invasive imaging is crucial for stroke prevention. Research data have demonstrated promising results, showing that the use of AI in plaque imaging enhances the accuracy of plaque risk stratification and may, in the future, be used to develop prognostic models to assess the risk of CIE and therefore support decisions on revascularization [78,79]. These findings suggest a possible role for miRNA assessment as noninvasive biomarkers that could become an additional tool incorporated into models for CAD diagnostics, monitoring, and prognostication, thereby improving preventive therapeutic approaches for CIEs.

The major advantage of using miRNAs as biomarkers is the ability to evaluate the expression of specific fragments using quantitative reverse transcriptase-polymerase chain reaction (qRT-PCR), the standard technology for detecting and comparing RNA levels with high specificity and sensitivity [48]. However, despite promising research on the potential of miRNAs as biomarkers for CAD and other pathologies, limitations have slowed the clinical application of circulating miRNAs [76]. From a technical standpoint, miRNAs may have the optimal biochemical properties to serve as readily accessible biomarkers. They are highly stable and have a long half-life in biological samples. The analyses require no special handling and can be performed at relatively low cost using standard, readily available techniques. Nonetheless, implementation of miRNA assessment in everyday practice requires additional efforts to overcome current methodological, technical, and analytical weaknesses. Available studies often yield inconsistent results due to a lack of standardization in specimen collection and processing, sample handling, miRNA quantification, and data analysis. Interpreting the current body of evidence poses a significant challenge due to a large number of animal model protocols. The lack of consensus on methodologies for miRNA quantification, faulty protocol designs that rely on limited use of large, independent cohorts, and a lack of replication of results are the main limiting factors in the clinical application of these biomarkers [80].

It is crucial to highlight that conflicting results from some studies do not necessarily indicate research pitfalls. Numerous studies report that the role of miRNAs in CAD etiology and progression may be context-dependent, varying across the disease course and among targeted cells. Upregulation of miR-21 in macrophages may exert a protective effect by promoting cholesterol efflux and inhibiting hyperlipidemia-induced inflammation [18]. In ECs, miR-21 may act as both a protective and a pathological factor, depending on the hemodynamic context. In VSMCs at the early stage of the disease, miR-21 may induce proatherotic proliferation and stabilize atherosclerotic plaques by reinforcing the fibrous caps in later stages of CAD. The conflicting results regarding miR-145 expression in the course of carotid atherosclerosis may also be time-dependent. While it is reported to regulate VSMC differentiation and inhibit neointimal formation, it may accelerate inflammation at advanced stages and is overexpressed in unstable atherosclerotic plaques [81]. miR-155 has been reported to promote macrophage foam cell formation, increase vascular inflammation, and inhibit inflammatory pathways by targeting MAP3K10 during early lesion development [82]. It should be noted, however, that the complexity of miRNA regulatory networks may raise significant concerns regarding not only their diagnostic but also potential future therapeutic use [83]. The beneficial features of miRNAs may be significantly limited due to their pluripotential mechanism of action. Therefore, miRNA signatures may be confounded by multiple comorbidities. Regarding therapeutic qualities, because a single miRNA can target hundreds of mRNAs, the knockout or inhibition of a key regulator in one process may cause unexpected and complex side effects. Their “multifunctional” qualities may indicate that a particular miRNA may play an advantageous therapeutic role in atherosclerosis but an essential homeostatic or regulatory role in other organs. Expanding knowledge of the complex mechanisms underlying miRNA function is a hallmark of progress in this area. It is necessary to critically analyze research results and design future study protocols that fully reflect miRNAs’ gene-regulating properties and, consequently, their diagnostic potential. Before miRNAs can be used for diagnostics, prognostication, and treatment in CAD, validation of identified miRNA biomarkers in multicenter comparative studies involving large patient cohorts is crucial.

Table 1 summarizes the role of miRNAs in carotid stenosis.

## 4. Conclusions and Future Perspectives

Research data confirm that miRNA expression is strongly correlated with various processes underlying plaque formation and the progression of carotid stenosis. These agents have a strong potential to become novel diagnostic tools and future therapeutic targets. Integrating multi-miRNA panels with standard laboratory assessments and neuroimaging, using AI-based protocols, could enhance predictive accuracy and enable the development of diagnostic and prognostic algorithms that may facilitate personalized treatment decisions for patients with CAD.

## Figures and Tables

**Table 1:** Role of miRNAs in carotid atherosclerosis.

CAD Stage	miR	Biological Function
**Endothelial dysfunction**	miR-21	under stress conditions—upregulated and contributes to a maladaptive inflammatory endothelial transformation, upregulation of endothelial adhesion molecules, and chemokine under normal hemodynamic conditions, plays a protective role in maintaining endothelial homeostasis by reducing endothelial apoptosis and promoting NO-mediated vasoprotection
	miR-125a-5p	plays a critical role in protecting brain endothelial barrier function cells with increased miR-125a-5p expression form thicker, more continuous junctional complexes of VE-cadherin and zona occludens-1
	miR-92a	upregulation by oxidized low-density lipoproteins enhances endothelial activation activation is flow-dependent, limited to low shear stress, atheroprone areas
	miR-200c	positively correlated with inflammatory markers, including monocyte chemoattractant protein-1, COX-2, IL-6, MMP-1, MMP-9 negatively correlated with protective agents indicated by ZEB1, eNOS, FOXO1, SIRT1
	miR-143/miR-145	responsible for the interaction between smooth muscle cells and endothelial cells determine VSMC phenotypic switching, and are essential for VSMC differentiation
**VSMCs proliferation and differentiation**	miR-195-5p	promotes VSMC proliferation and migration inhibits the protective role of LINC01088 on HAECs proliferation, migration, and apoptosis.
	miR-206	downregulation is correlated with reduced relative VSMCs survival rate high blood glucose and lipid levels decrease miR-206 expression by inducing metabolic inflammation and functional changes in VSMCs
	miR-361-5p	promotes VSMCs proliferation and migration by targeting the tissue inhibitor of TIMP4 gene inhibition of TIMP4 results in atherosclerotic plaque deposition
	miR-532-3p	expression is inversely correlated to IM thickness over-expression inhibits the proliferation and migration in VSMCs
	miR-143	maintains the contractile stable phenotype of VSMCs downregulation is associated with increased proliferation and migration of VSMCs
	miR-145	regulates the phenotype of highly expressed VSMCs inhibits the transition of VSMCs to synthetic, proliferative, or migratory cells
	miR-210	directly targets the APC gene, promoting VSMCs’ survival and reducing apoptosis
**Monocyte recruitment and foam cell formation**	miR-22-3p	reduces the inflammatory response by inducing the anti-inflammatory macrophage phenotype Suppresses NLRP3 inflammasome activation via the JAK1 gene
	miR-155	amplifies inflammation by inhibiting STAT signaling and promoting pro-inflammatory polarization directly represses Bcl6, accelerating foam cell formation and inducing atherosclerotic plaque development
	miR-126	Improves efferocytosis and plaque stability, facilitating anti-inflammatory phenotype switching by targeting the vascular endothelial growth factor A
	miR-223/miR-146a/miR-218-5p cluster	reduce the signaling of main initiator of pro inflammatory cytokine release—TLR4 inhibit the secretion of TNF-α, IL-6, and IL-12
	miR-127-3p	overexpression decreases content of UFAs, increased content of acetyl CoA and increased level of oxidative phosphorylation miR-127-3p-overexpressed macrophages exhibit increased proliferation capacity and cholesterol accumulation
	miR-27 and miR-329	downregulate monocytic ABC protein expression inhibit elimination of excess cholesterol and forming of high-density lipoprotein
**Plaque instability**	miR-146a	upregulated in peripheral blood monocytes from patients with vulnerable plaques
	miR-155	elevated in pro-inflammatory macrophages found in atherosclerotic lesions
	miR-206 and miR-638	act as plaque-stability protectors reduce the risk of ischemic stroke recurrence
	miR-124-3p, miR-134-5p, miR-34a-5p, miR-375, and miR-133b	levels significantly altered in soft and echolucent plaques
	miR-16-5p and miR-1-3p	overexpression characteristic of ulcerated plaques
	miR-1-3p	high levels are an independent risk factor for CVD and MI
	miR-133b	high levels positively associated with the incidence of CIE
	miR-23a-5p, miR-320a, miR-2110, and miR-134-5p	upregulated in vulnerable plaques
	miR-4439	downregulated in vulnerable plaques

**NO**—Nitric oxide, **COX-2**—cyclooxygenase-2, **IL-6**—interleukin 6, **MMP-1**—matrix metalloproteinase 1, **MMP-9**—matrix metalloproteinase 1, **ZEB1**—zinc finger E-box binding homoeobox 1, **eNOS**—endothelial nitric oxide synthase, **FOXO1**—forkhead boxO1, **SIRT1**—Sirtuin1, **VSMCs**—vascular smooth muscle cells, **LINC01088**—long noncoding RNA 01088, **HAECs**—human aortic endothelial cells, **TIMP4**—tissue inhibitor of matrix metalloproteinase 4 gene, **IM**—intima media, **APC**—adenomatous polyposis coli gene, **NLRP3**—NLR family pyrin domain containing 3 inflammasome, **JAK1**—Janus Kinase 1 gene, **STAT**—signal transducer and activator of transcription, **Bcl6**—B-cell lymphoma 6 gene, **TLR4**—Toll-like receptor 4, **TNF-α**—tumor necrosis factor alpha, **IL-12**—interleukin 12, **UFAs**—unsaturated fatty acids, **ABC**—ATP-binding cassette protein, **CVD**—cardiovascular disease, **MI**—myocardial infarction, **CIE**—cardiovascular ischemic event.

## Data Availability

No new data were created or analyzed in this study. Data sharing is not applicable to this article.

## Abbreviations

The following abbreviations are used in this manuscript:

CAD	Carotid atherosclerotic disease
CAS	Carotid artery stenosis
CIE	Cardiovascular ischemic event
ECs	Endothelial cells
hs-CRP	High-sensitivity C-reactive protein
IM	Intima media
Lp-PLA2	Lipoprotein-associated phospholipase A2
miRNA	MicroRNA
NO	Nitric oxide
qRT-PCR	Quantitative reverse transcription polymerase chain reaction
VSMCs	Vascular smooth muscle cells
